# Lessons from In Utero and Postnatal Exposure to Pesticide DDT: Mechanisms of Chromaffin Cell Vulnerability and Altered Catecholamine Homeostasis

**DOI:** 10.3390/ijms27188323

**Published:** 2026-09-18

**Authors:** Nataliya V. Yaglova, Victor A. Tutelyan, Inna Yu. Tarmaeva, Sergey S. Obernikhin, Dmitriy B. Nikityuk

**Affiliations:** 1 Federal Research Centre of Nutrition, Biotechnology and Food Safety, Ustinsky Passage 2/14, Moscow 109240, Russia; tutelyan@ion.ru (V.A.T.); tarmaeva@ion.ru (I.Y.T.); ober@mail.ru (S.S.O.); dimitrynik@mail.ru (D.B.N.); 2 Institute of Clinical Medicine Named After N.V. Sklifosovsky, I.M. Sechenov First Moscow State Medical University (Sechenov University), 8-2 Trubetskaya Str., Moscow 119991, Russia

**Keywords:** endocrine-disrupting chemical, DDT, neuroendocrine cells, chromaffin cells, adrenal medulla, epinephrine, developmental program, regeneration capacity

## Abstract

Dichlorodiphenyltrichloroethane (DDT) remains one of the most emblematic and enduring persistent organic pollutants of the 20th century. Although prohibited for agricultural use in most industrialized nations during the 1970s–1980s on the grounds of its persistence, bioaccumulation, and carcinogenicity, DDT was reintroduced by the World Health Organization in 2006 for indoor residual spraying against vector-borne diseases. Consequently, its environmental burden continues to be replenished rather than diminished. Biomonitoring surveys confirm the near-ubiquity of DDT residues in human populations. Low-dose human exposure to DDT occurs predominantly through contaminated food. Its main health risk is the ability of DDT to disrupt endocrine function. Numerous reports show negative effects of DDT on the reproductive system, the thyroid gland, and immune defense. Affection of the nervous system is sufficiently less studied. This article presents known mechanisms of endocrine disruption and summarizes current data on the disruption of the adrenal medulla by low-dose exposure to DDT. The review focuses on the three main aspects of chromaffin cell biology. The first one is endocrine function, including synthesis of catecholamines, secretory machinery and release of catecholamines into circulation. The second is embryonic and postnatal development of the adrenal medulla, and the third one is physiological cell renewal and maintenance of cell population, a novel enigmatic aspect of endocrine disruption. In conclusion, the authors present integrated mechanisms of disruption and indicate gaps in knowledge and directions for further investigations.

## 1. Introduction

The nervous and endocrine systems regulate all visceral functions via complex interacting mechanisms. Disorders of their development as well as interference in the interaction of nervous and endocrine control inevitably lead to severe health consequences. The adrenal medulla, predominantly composed of chromaffin cells, serves as the primary effector of the sympatho-adrenal system and the main source of circulating catecholamines, particularly epinephrine [1]. Derived embryologically from the neural crest, chromaffin cells exhibit a unique neuroendocrine phenotype, functioning as modified sympathetic neurons that release catecholamines directly into the systemic circulation in response to physiological or psychological stressors [2]. The precise regulation of chromaffin cell development, differentiation, and functional activity is critical for maintaining cardiovascular homeostasis, metabolic balance, the organism’s “fight-or-flight” response and adaptation to any stress factor [3].

The ontogenesis of chromaffin cells is a highly orchestrated process that relies on a complex interplay of intrinsic genetic programs and extrinsic microenvironmental cues. A defining feature of adrenal medulla development is the corticomedullary cross-talk: high local concentrations of glucocorticoids, delivered via the intra-adrenal portal blood system from the adrenal cortex, are essential for the terminal differentiation of chromaffin cells, specifically inducing the expression of phenylethanolamine N-methyltransferase (PNMT), the enzyme responsible for converting norepinephrine to epinephrine [4]. Because of this intricate developmental trajectory and their dependence on local paracrine signaling, chromaffin cells are highly susceptible to perturbations during embryonic and early postnatal life, aligning with the Developmental Origins of Health and Disease (DOHaD) concept [5]. Because chromaffin cells are both nervous and endocrine, they may be the most vulnerable to the action of endocrine disruptors. Endocrine-disrupting chemicals are exogenous substances that interfere with the synthesis, secretion, transport, action, or elimination of natural hormones [6]. They include hundreds of substances with different chemical structures and routes of exposure.

In terms of frequency and duration of exposure, the most dangerous substances are those found in food products, as exposure to them can last a person’s entire life and exert the most pronounced effects on organ development and function. Endocrine disruptors enter food products from the environment (e.g., residual amounts of agrochemicals introduced into the soil or previously present in it) or packaging, or arise during food processing and preparation [7,8]. The former pose the greatest risk because, as persistent pollutants, they cannot be eliminated from raw materials or finished food products.

In recent decades, the ubiquitous presence of endocrine-disrupting chemicals in the environment has emerged as a major global health concern. While the reproductive system and thyroid axis have been extensively studied in the context of EDC exposure, the impact of these chemicals on the sympatho-adrenal axis and, specifically, on adrenal chromaffin cells, remains significantly underexplored [9,10,11,12]. The objective of this review is to summarize data on the changes caused by low-dose exposure to the most persistent in the environment endocrine disruptor, dichlorodiphenyltrichloroethane (DDT), in the development and function of chromaffin cells in order to draw useful lessons from the mechanisms and manifestations of DDT action for future research on neuroendocrine disruption.

## 2. DDT: Persistence, Exposure and Endocrine-Disrupting Properties

DDT is a well-known organochlorine chemical with excellent insecticidal properties. It acts as a contact insecticide affecting the nervous system. DDT binds to voltage-gated sodium channels in the peripheral and central nervous systems, stabilizing them in the open state and causing repetitive, uncontrolled nerve firings, resulting in spasm and eventual death [13]. Low production costs, high efficiency and relatively low acute toxicity for humans and animals led to the widespread use of DDT in both agriculture and everyday life. Half a million tons of DDT were used annually around the world in 1950–1970s, with 70–80% of that being used in agriculture. This resulted in its ubiquitous dissemination and persistence due to its long half-life and resistance to biodegradation. In the 1970s–1980s, the use of DDT in agriculture was banned by the Stockholm Convention on Persistent Organic Pollutants and later in vector-borne disease control. In 2006, the World Health Organization resumed the use of DDT to control malaria and leishmaniasis vectors in 12 countries around the world, as insecticides used instead of DDT proved ineffective, leading to a significant increase in these diseases. DDT is currently found in all ecosystems on the planet, including the oceans. The resumption of DDT use has resulted in a slower rate of decline in its concentrations in water and soil [14,15]. DDT not only pollutes soils and waters but also accumulates in the food chain [14]. This explains its presence in foods of plant and animal origins. DDT levels in food are regulated by international and national regulations, but in reality, they reflect its distribution in plants and animals, with higher levels in animal products and lower levels in plant products. Maximal permissible levels for DDT in food range, on average, from 50 in plant products up to 200–400 μg/kg in meat and fish, and a provisional tolerable daily intake of DDT and its associated compounds is 0.01 mg/kg bw [15].

**Figure 1:**
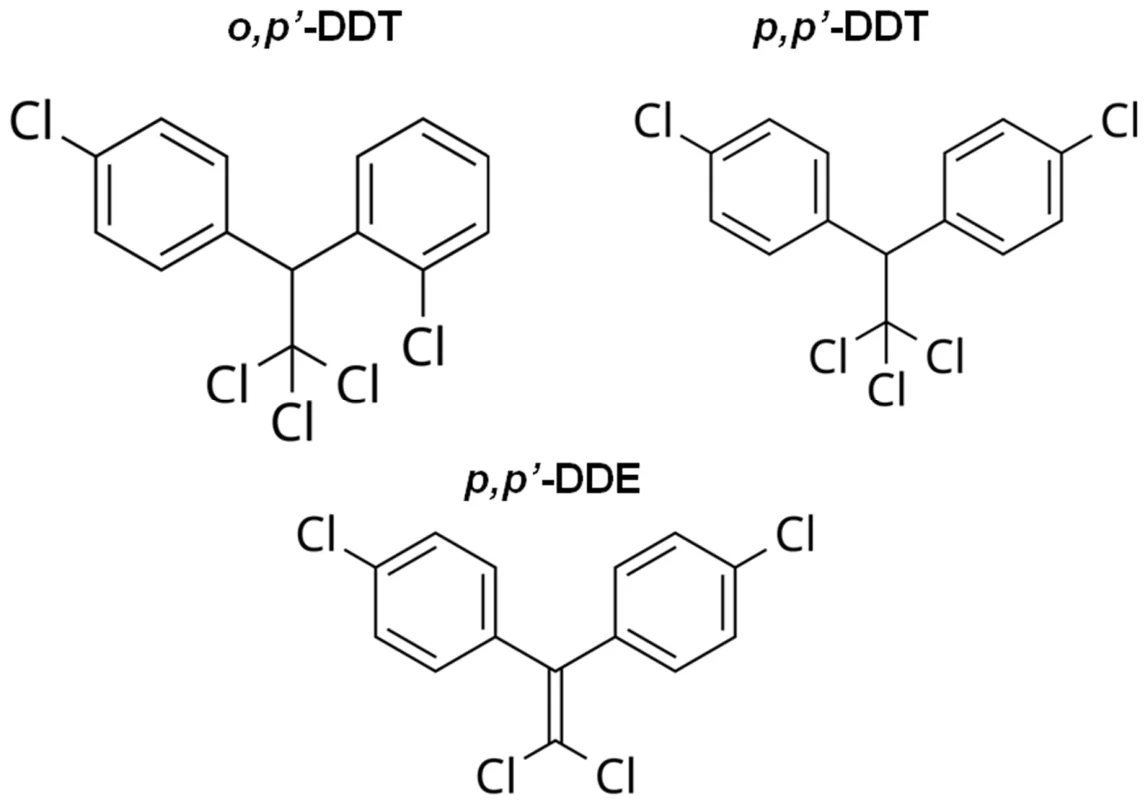
Structure of DDT isomers and its key metabolite.

DDT is a small molecule that has two isomers (Figure 1). The most common is the *p,p’*-isomer. Due to low molecular weight and high lipophilicity, both isomers are easily absorbed into the intestinal lymphatic system and penetrate blood–tissue barriers. DDT metabolites accumulate in lipid-rich tissues, mainly adipose tissue, and are stored for a long period of time. DDT and its main metabolite dichlorodiphenyldichloroethylene (DDE) remain in the human body for years, with a half-life of 3 to 6 years for DDT and roughly 6 to 12 years for DDE [16].

Exposure to DDT begins in early life since it crosses the fetal–placental barrier and accumulates in breast milk. DDT and its primary metabolite DDE routinely accumulate in human placental tissues, with reported levels varying widely based on geographic location, local dietary habits, and historical use. Typical concentrations range from low parts-per-billion (pg/g or ng/g lipid weight) up to several micrograms per kilogram (µg/kg), demonstrating continuous transplacental transfer [17,18,19]. Despite the decline in DDT and its metabolite concentrations in breast milk in recent decades, its presence is detected in nearly 100% of samples, regardless of region of residence [20,21,22]. Global monitoring shows that pronounced geographic differences remain in DDT concentrations in human milk. The lowest levels are found in Europe, central regions of the USA, Russia, Latin America, and Australia, and the highest ones are found in Southeast Asia, especially in India [21,22,23]. Placental and lactational transfer poses the greatest risk to human health because it occurs during periods of greatest vulnerability. Prenatal and postnatal exposures to low doses of DDT are associated with changes in the developmental program of the endocrine and immune systems and have significant long-term consequences [24,25,26,27]. Along with developmental and functional disruption, DDT has been shown to induce the epigenetic transgenerational inheritance of disease [28,29]. Thus, low-dose exposure to DDT poses a serious threat to the health of current and future generations and requires a systematic approach to identifying its consequences.

DDT is one of the compounds for which endocrine disruptor activity was first established. Early studies linked the disruptive effects of DDT to its ability to bind to nuclear receptors. DDT and its metabolites are considered potent estrogen receptor agonists and progesterone receptor and androgen receptor antagonists [30,31]. Activation of estrogen and inhibition of androgen receptors increase estrogen signaling and make it predominant. Developmental exposure to DDT results in accelerated maturation of female organisms and disrupted masculinization in males. Numerous studies have revealed higher incidence of precocious puberty, malformations and impaired function of the reproductive system in both sexes and higher risks of breast cancer in regions where DDT was used as a pesticide for crop protection or for vector disease control [32,33,34,35,36,37,38]. DDT has also been found to disrupt thyroid-stimulating hormone receptor signaling by inhibition of its activation and internalization [39,40]. The anti-thyroid action of DDT also includes inhibition of sodium/iodide symporter production, ensuring transportation of iodide ions into cells, and impaired thyroglobulin endocytosis and reduction of lysosome formation in thyroid cells, necessary for thyroglobulin cleavage [12,27]. Thus, DDT creates a hormonal background with a predominance of estrogens and a deficiency of androgens and thyroid hormones, which leads to an imbalance in the hormonal signaling that regulates cell proliferation, differentiation, and apoptosis.

Besides interacting with nuclear receptors, DDT and its metabolites evoke mitochondrial dysfunction. They disrupt the electron transport chain and lead to excessive production of reactive oxygen species [41]. DDT and DDE were shown to affect Complex II (succinate dehydrogenase) and Complex V (ATP synthase) of the electron transport chain, impairing cell respiration and production of ATP [42,43]. Lack of energy and oxidative stress are well-known triggers that activate apoptosis pathways. Induction of apoptosis after DDT exposure was reported in various cell types, not just endocrine cells [44,45,46,47,48,49,50].

**Figure 2:**
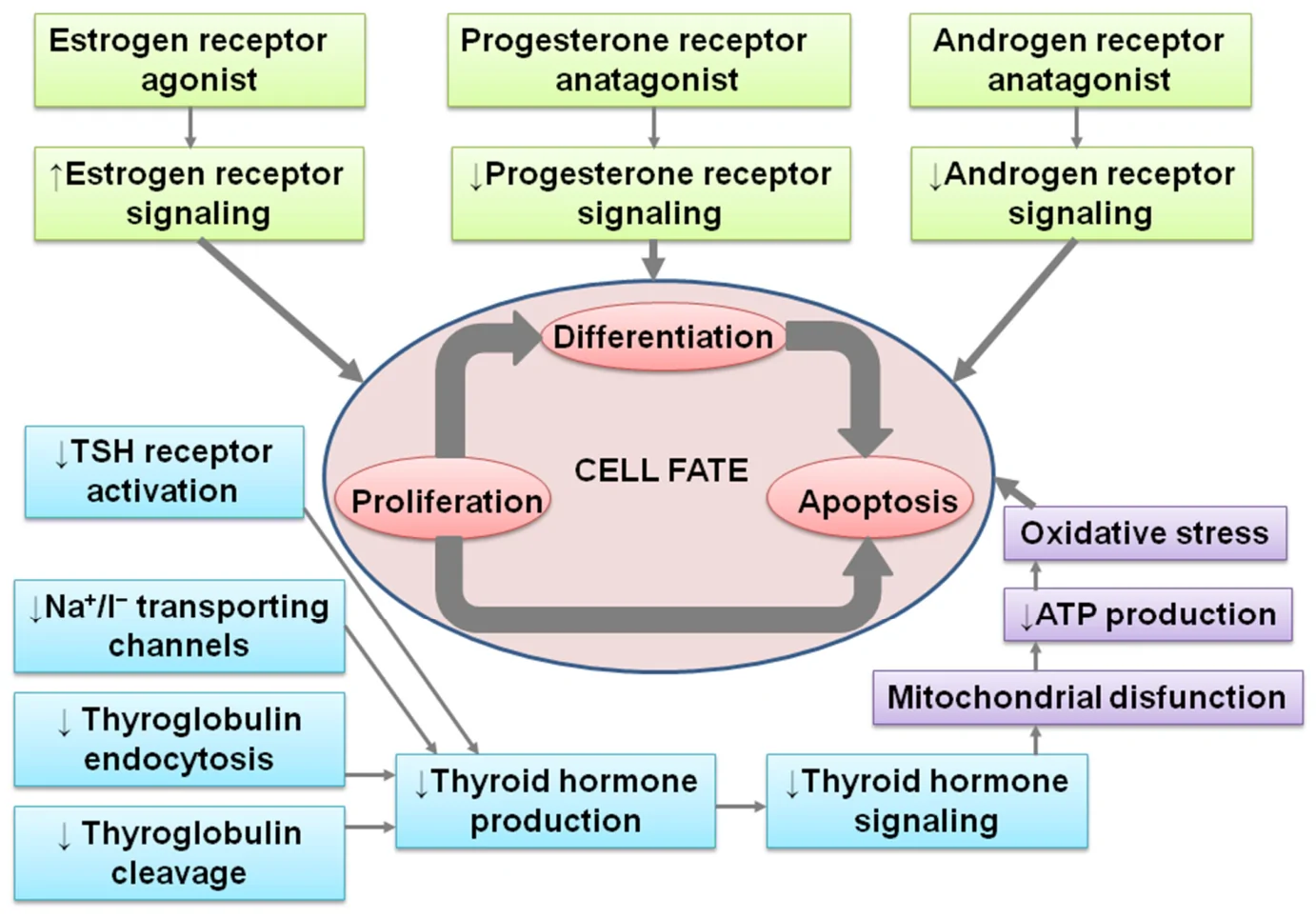
Identified mechanisms of DDT disruptive action on cell development and function.

Thus, DDT alters the balance of thyroid and gonadal hormone signaling, regulates morphogenetic processes, affects ion channels, impairs mitochondrial function and, consequently, dysregulates organelle function and the secretory machinery of endocrine cells. It is clear that the molecular and cellular mechanisms of DDT action can negatively influence any cell type, including nervous system cells, in two main ways. The first is by directly interfering with cell functioning. The second is by impacting cell proliferation, differentiation and apoptosis (Figure 2).

## 3. Disruption of Chromaffin Cell Function

The neurotoxic potential of DDT within the central nervous system—its capacity to perturb neuronal excitability, impair cognition, and induce neurobehavioral deficits—is comparatively well characterized [51,52,53], and epidemiological associations have even been drawn between DDT metabolite accumulation in brain tissue and Parkinson’s disease in agricultural workers occupationally exposed to higher doses of pesticides [54]. By contrast, the consequences of low-dose DDT exposure for the peripheral nervous system and, more specifically, for neuroendocrine regulatory circuits remained a conspicuous lacuna.

Within this underexplored domain, the adrenal medulla and its constituent chromaffin cells represent a target of exceptional vulnerability and physiological importance. Chromaffin cells are derivatives of the neural crest, arising from Schwann cell precursors that migrate along preganglionic sympathetic axons to colonize the adrenal anlage during embryonic development [1,55,56]. They share extensive transcriptional, structural, and functional homology with postsynaptic sympathetic neurons, deploying an analogous catecholamine biosynthetic machinery and an equivalent regulated exocytotic apparatus [2]. This neuronal lineage identity confers upon chromaffin cells a heightened susceptibility to lipophilic neurotoxicants that partition readily into their membranes and organelles. Functionally, the adrenal medulla furnishes up to 80% of the circulating epinephrine pool, positioning it as the principal humoral effector of the sympatho-adrenal stress axis [57]. Any xenobiotic-induced compromise of chromaffin cell integrity therefore threatens systemic catecholamine homeostasis. Importantly, whereas the sensitivity of the adrenal cortex to both the toxic and disruptive actions of DDT and the differential vulnerability of its three steroidogenic zones has received considerable attention, the adrenal medulla has remained comparatively neglected [58,59]. Emerging experimental evidence nonetheless indicates that developmental low-dose DDT exposure suppresses adrenomedullary epinephrine output, disrupting catecholamine synthesis and/or secretion [60]. In this regard, later in the article, we will consider the mechanisms of synthesis and release of catecholamines and how DDT can disrupt these processes.

### 3.1. Synthesis and Secretion of Catecholamines in Chromaffin Cells

#### 3.1.1. Biosynthesis and Storage of Catecholamines

**Figure 3:**
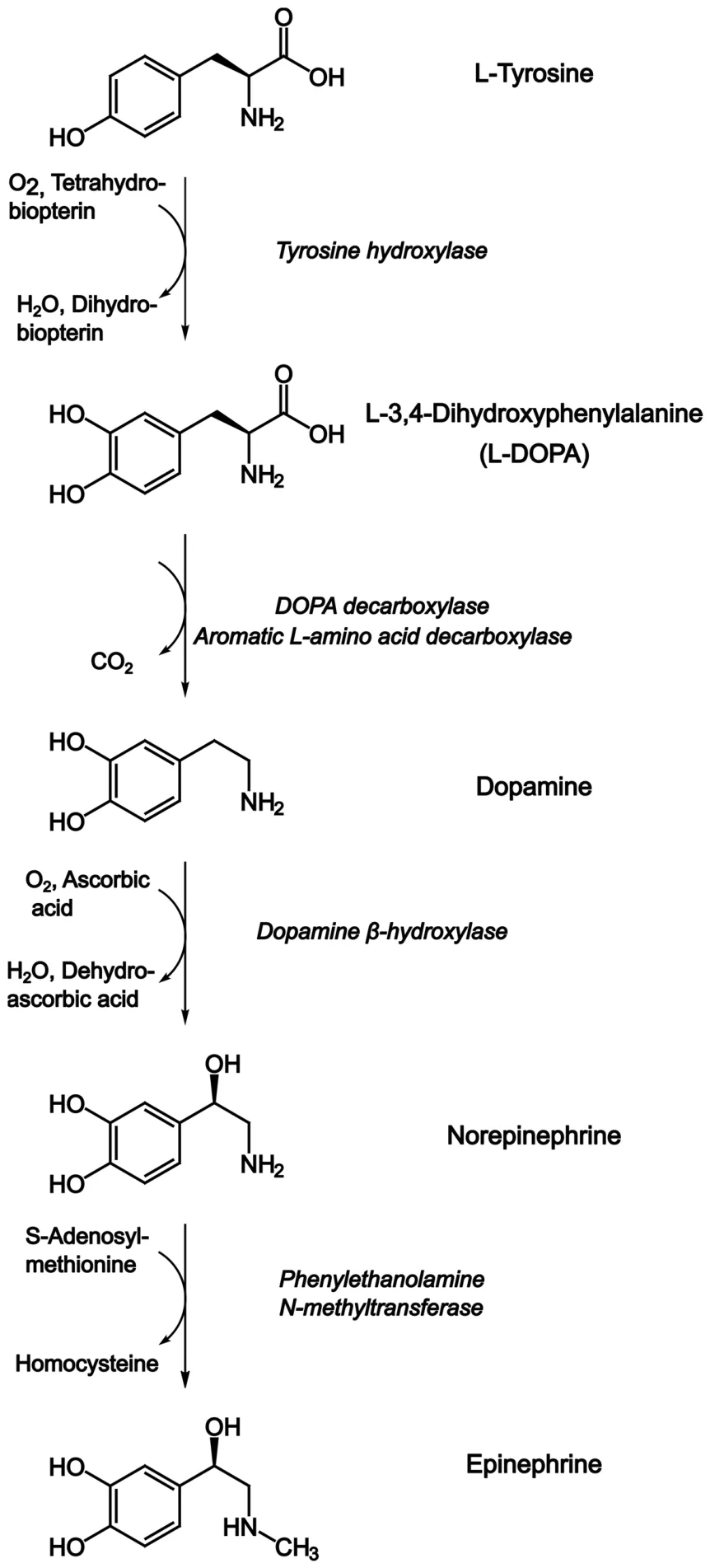
The main steps of catecholamine synthesis.

The biosynthesis of catecholamines in chromaffin cells follows a well-characterized enzymatic pathway initiated by the uptake of the amino acid tyrosine from the circulation via the L-type amino acid transporter (LAT1) [61]. The first and rate-limiting step is the hydroxylation of tyrosine to L-3,4-dihydroxyphenylalanine (L-DOPA), catalyzed by tyrosine hydroxylase (Figure 3). Tyrosine hydroxylase is a tetrahydrobiopterin- and iron-dependent monooxygenase whose activity is tightly regulated by phosphorylation at multiple serine residues (Ser19, Ser31, Ser40) through the cAMP-dependent protein kinase A (PKA), protein kinase C (PKC), and Ca^2+^/calmodulin-dependent protein kinase II (CaMKII) pathways [62]. This phosphorylation-dependent regulation is critical for the rapid adjustment of catecholamine output in response to sustained stress. Long-term regulation of tyrosine hydroxylase involves tyrosine hydroxylase gene expression. The tyrosine hydroxylase gene promoter contains numerous transcription factor-binding sites that permit mRNA levels to be regulated by Ca^2+^, glucocorticoids, neuronal activity, hypoxia, and stress [63,64]. However, a number of studies have reported major discrepancies between trigger-induced changes in tyrosine hydroxylase gene transcription, tyrosine hydroxylase mRNA, and tyrosine hydroxylase protein [65,66,67,68]. These discrepancies suggest that post-transcriptional mechanisms also play an important role in regulating tyrosine hydroxylase expression in response to stress and other stimuli. Tyrosine hydroxylase activity strictly depends on the amounts of dopamine, epinephrine and norepinephrine in cells. End-product catecholamines bind directly to the enzyme and slow it down by competing with its natural cofactor, tetrahydrobiopterin. The feedback inhibition by end-products plays the principal role in tyrosine hydroxylase control [62]. Another mechanism of enzyme inhibition is tyrosine hydroxylase degradation by the ubiquitin–proteasome coupled pathway [64].

L-DOPA is subsequently decarboxylated to dopamine by aromatic L-amino acid decarboxylase, a pyridoxal phosphate-dependent enzyme that is not rate-limiting under physiological conditions [62]. Dopamine is then transported into chromaffin granules via the vesicular monoamine transporter 1 (VMAT1), where it is converted to norepinephrine by dopamine β-hydroxylase (DBH), a copper- and ascorbate-dependent monooxygenase co-packaged with catecholamines, ATP, and opioid peptides within the granule lumen [69].

The final step, specific to adrenal chromaffin cells, is the N-methylation of norepinephrine to epinephrine. The reaction is catalyzed by PNMT, utilizing S-adenosylmethionine as a methyl donor. PNMT expression is strictly dependent on the high local concentrations of glucocorticoids delivered via the intra-adrenal portal system, making epinephrine synthesis a hallmark of the adrenal medullary phenotype [70]. Pituitary ablation, which prevents corticosteroid production, was shown to reduce PNMT expression in rats. Expression can be fully restored to normal levels, but not beyond, through corticosteroid replacement, indicating the impact of glucocorticoids on catecholamine synthesis [71]. Corticosteroid hormones control PNMT activity post-translationally by indirectly preventing PNMT degradation [72]. Glucocorticoids mediate and sustain the expression of S-adenosylmethionine metabolic enzymes, including methionine adenosyltransferase and *S*-adenosylhomocysteine hydrolase. Their activity provides sufficient S-adenosylmethionine required both for catalysis and stabilization of PNMT by masking proteolytically vulnerable regions of the protein [70]. Another mechanism of glucocorticoid control involves regulating the expression of PNMT mRNA [73,74].

Synthesized catecholamines are stored in large dense-core vesicles known as chromaffin granules. Epinephrine and norepinephrine are stored in different granules. Chromaffin granules have an average diameter of 200–400 nm and contain a highly concentrated internal matrix composed of catecholamines (up to 0.5 M), ATP, chromogranins, neuropeptides, and dopamine β-hydroxylase [75]. The acidic interior of the granule (pH ~5.5), maintained by a vacuolar-type H^+^-ATPase (V-ATPase), provides the electrochemical driving force for VMAT1-mediated uptake of monoamines in exchange for two protons [76].

Chromogranins, the most abundant soluble proteins in chromaffin granules, play a dual role: they serve as pro-hormone precursors for bioactive peptides (e.g., vasostatin, pancreastatin) and function as a granule matrix organizer, facilitating the condensation of catecholamines with ATP and calcium into an osmotically inactive form suitable for high-density storage [77]. The biogenesis of chromaffin granules involves the trans-Golgi network, where cargo proteins including dopamine β-hydroxylase and VMAT1 are sorted into immature secretory granules that undergo maturation through acidification and proteolytic processing [78,79].

#### 3.1.2. Secretion of Catecholamines

Adrenal chromaffin cells, like other cell types containing secretory granules, are known to secrete synthesized products through exocytosis mediated by kiss-and-collapse and kiss-and-run mechanisms, piece-meal degranulation, or molecular secretion [80,81]. Exocytosis, the process in which the granule membrane merges with the plasma membrane, opening a fusion pore to release cargo into the extracellular space, is considered the main route of catecholamine discharge.

The adrenal medulla is innervated by preganglionic sympathetic cholinergic fibers that release acetylcholine onto nicotinic acetylcholine receptors of chromaffin cells, predominantly the α3β4 subtype [82,83]. Activation of nicotinic acetylcholine receptors leads to rapid Na^+^ influx and membrane depolarization, which in turn opens voltage-gated calcium channels (VGCCs), primarily of the N-, L-, and P/Q-types in bovine and rodent chromaffin cells [84]. The resulting calcium influx triggers the exocytotic fusion of chromaffin granules with the plasma membrane. Raised concentrations of extracellular calcium ions shift the preferred mode of exocytosis to the kiss-and-run mechanism in a calcium-concentration-dependent manner [85].

The molecular machinery of exocytosis in chromaffin cells has been extensively characterized using these cells as a model system for regulated secretion. The core SNARE complex consists of syntaxin-1 and SNAP-25 on the plasma membrane (t-SNAREs) and synaptobrevin-2 (VAMP2) on the granule membrane (v-SNARE) [86]. The calcium sensor synaptotagmin-1, anchored to the granule membrane, binds calcium ions with low affinity and high cooperativity, triggering the final fusion step within milliseconds of calcium entry [87]. Seminal work by Neher and colleagues, using patch-clamp capacitance measurements in bovine chromaffin cells, revealed that exocytosis occurs in at least two kinetically distinct phases: a rapidly releasable pool of granules docked at the plasma membrane that fuse within tens of milliseconds and a slowly releasable pool that requires priming and replenishment over seconds to minutes [88,89]. This biphasic release is physiologically significant, as it allows chromaffin cells to provide both an immediate catecholamine surge and a sustained secretory response during prolonged stress.

Following exocytosis, the granule membrane is retrieved via clathrin-mediated endocytosis or bulk endocytosis, and granules are re-acidified and reloaded with catecholamines in a process termed “kiss-and-run” or full-collapse retrieval, depending on the stimulus intensity [90,91]. This granule recycling is essential for maintaining the secretory capacity of chromaffin cells during repeated or sustained stimulation, and its disruption represents a potential target for xenobiotic toxicity.

Cromaffin cell secretion is modulated by multiple autocrine and paracrine mechanisms. Nicotine and other nicotinic agonists produce a biphasic response: initial stimulation followed by receptor desensitization, a phenomenon of considerable toxicological relevance [92]. Opioid peptides co-released from chromaffin granules activate μ-opioid receptors on the same cells, providing negative feedback that inhibits further secretion of catecholamines [93].

### 3.2. Vulnerability to Endocrine Disruption of Chromaffin Cell Secretion

The physiological framework outlined above identifies multiple vulnerable targets for endocrine-disrupting chemicals: (i) the enzymatic machinery of catecholamine biosynthesis, particularly tyrosine hydroxylase, whose activity is sensitive to oxidative modification, and PNMT, whose secretion is controlled by adrenocortical cell hormones; (ii) VMAT1 function and granule acidification, which can be compromised by mitochondrial toxins; (iii) VGCCs, which are direct molecular targets of several organochlorine pesticides, including DDT; and (iv) SNARE-dependent fusion machinery, which requires precise calcium microdomain signaling [94]. The subsequent sections will examine how DDT and its metabolites specifically interfere with these processes.

### 3.3. Identified Mechanisms of DDT Disruption of Adrenomedullary Secretion

In vivo studies show that exposure to low doses of DDT within the maximum permissible levels in food causes a gradual decrease in the blood levels of both epinephrine and norepinephrine [60]. Notably, a decrease in blood epinephrine, indicative of adrenomedullary chromaffin cell dysfunction, was registered in animals exposed prenatally and postnatally as well as only postnatally. The only difference was the onset time of the decline. When exposure began prenatally, the decline was more rapid. Thus, the period of ontogeny when exposure to DDT begins is not critical.

Immunohistochemical evaluation of the enzymes catalyzing catecholamine synthesis in adrenal chromaffin cells revealed a progressive reduction in cytoplasmic tyrosine hydroxylase immunoreactivity in exposed animals and emergence of a nearly tyrosine hydroxylase-negative subpopulation of chromaffin cells that was essentially absent from control medullae [95,96]. Because tyrosine hydroxylase is simultaneously the rate-limiting enzyme of catecholamine biosynthesis and the canonical marker of terminal chromaffin differentiation, its loss may signify enzymatic downregulation in differentiated cells, failure of terminal differentiation, or dedifferentiation of mature cells.

Low-dose developmental exposure to DDT suppresses tyrosine hydroxylase production in a dose- and window-dependent manner. This effect is most pronounced when exposure begins postnatally, which yields up to 23–28% tyrosine hydroxylase-negative cells in the rat adult medulla [95]. Since tyrosine hydroxylase catalyzes the first step of catecholamine production, its deficit explains the decline in both epinephrine and norepinephrine levels. Unlike epinephrine, norepinephrine in the blood pool originates from sympathetic neurons. The reported combined decrease in blood levels of epinephrine and norepinephrine indicates that DDT exerts disruptive action on catecholamine-producing cells of different types. Surprisingly, the decline of blood norepinephrine levels in exposed animals was more profound than epinephrine levels [60]. This suggests involvement of additional mechanisms possibly associated with less transmitter spillover in the synaptic cleft due to enhanced reuptake. A distinctive feature of the inhibitory effect of DDT on the synthesis of tyrosine hydroxylase is its significant increase after puberty, regardless of whether exposure begins prenatally or postnatally [96].

Secretion of catecholamines from cells can also be a target for endocrine disruptors, as it is a multicomponent and often energy-intensive process. Catecholamine release proceeds primarily via calcium-dependent exocytosis of secretory granules, which requires the participation of mitochondria. Two functionally distinct mitochondrial populations, perinuclear and subplasmalemmal, have been identified in chromaffin cells; the latter directly support exocytosis by buffering local calcium transients at release sites [97,98,99]. Chromaffin cells of DDT-exposed rats showed reduced total mitochondrial number, with the loss disproportionately observed in the subplasmalemmal population, precisely the compartment that shapes the local calcium microdomains required for granule fusion [95,100]. Surviving mitochondria displayed matrix swelling and disrupted cristae. Their topography was displaced toward a perinuclear region [100,101]. The number of catecholamine-storing secretory granules and their electron density in the cytoplasm was likewise diminished [95,100]. All these findings are indicative of lowered catecholamine production and disrupted secretory machinery. An alternative secretory route, piecemeal degranulation, in which granule content dissolves and is released in molecular form without full exocytosis, is enhanced in DDT-exposed and aged chromaffin cells and appears to partially compensate for exocytotic failure [95].

## 4. Disruption of Chromaffin Cell Development

The known ability of DDT to bind nuclear receptors of steroid hormones suggests alterations in the balance of cell growth and differentiation in both embryonic and postnatal ontogeny.

### 4.1. Embryonic Development of Chromaffin Cells and Its Regulation

Adrenal chromaffin cells originate from the neural crest, a transient, multipotent cell population that arises at the border between the neural plate and the non-neural ectoderm during early embryogenesis [102]. They arise from neuron-associated multipotent cells, also known as Schwann cell precursors [102,103,104]. During embryonic development, chromaffin cell precursors undergo a complex migration pathway. They first migrate ventrally through the somites to form the sympathetic ganglia, and subsequently, a subset of these cells invades the developing adrenal cortex to form the adrenal medulla [105]. The migration of cells is guided by a combination of chemoattractant signals, including neuregulin-1 and its receptor ErbB3, as well as repulsive cues such as semaphorins and ephrins [102,106]. The precise timing and coordination of this migration are critical, as disruptions can lead to ectopic chromaffin cell clusters or hypoplasia of the adrenal medulla.

The differentiation of Schwann cell precursors into chromaffin cells begins at 11.5 days of human embryonic development, whereas in rats, chromaffin cell differentiation begins on the 18th day of the prenatal period and finishes on the 10th day of postnatal life [104,106,107].

The differentiation of neural crest-derived precursors into mature chromaffin cells is governed by a hierarchical network of transcription factors. Among the most critical are the paired-like homeodomain transcription factor PHOX2B, the basic helix–loop–helix factors MASH1 (also known as ASCL1) and HAND2, and the LIM-homeodomain factor ISL1 [108]. PHOX2B is considered a master regulator of autonomic neuron development and is essential for the survival and differentiation of chromaffin cell precursors; mutations in PHOX2B are associated with congenital central hypoventilation syndrome and Hirschsprung disease, conditions often accompanied by adrenal medullary dysfunction [109,110]. MASH1 and HAND2 play complementary roles in specifying the catecholaminergic phenotype. MASH1 is required for the initial commitment to the sympathetic lineage, while HAND2 is essential for the maintenance and expansion of chromaffin cell progenitors [111]. These transcription factors activate downstream targets, including tyrosine hydroxylase, dopamine β-hydroxylase, and the vesicular monoamine transporter VMAT1, which are necessary for catecholamine synthesis and storage [112].

A unique feature of adrenal chromaffin cell development is the dependence on local paracrine signals from the adjacent adrenal cortex. Unlike sympathetic ganglion neurons, which remain predominantly noradrenergic, adrenal chromaffin cells acquire the ability to synthesize epinephrine through the expression of PNMT [113]. This terminal differentiation step is strictly glucocorticoid-dependent. High concentrations of glucocorticoids, delivered via the intra-adrenal portal blood system that connects the adrenal cortex to the medulla, activate the glucocorticoid receptor in chromaffin cells, which directly binds to glucocorticoid response elements in the PNMT gene promoter, inducing its transcription [73]. This corticomedullary cross-talk ensures that epinephrine production is tightly coupled to the functional maturation of the adrenal gland and occurs predominantly during late fetal and early postnatal development in rodents, or during the third trimester of human pregnancy [114]. Disruption of this paracrine axis, whether through genetic ablation of the glucocorticoid receptor in chromaffin cells, surgical hypophysectomy, or exposure to endocrine-disrupting chemicals that alter adrenal steroidogenesis, results in a dramatic reduction in PNMT expression and a shift toward a predominantly noradrenergic phenotype [113].

### 4.2. Vulnerability to Endocrine Disruptors During Development

The developmental program of chromaffin cells, with its reliance on precise temporal and spatial coordination of migration, transcriptional regulation, and paracrine signaling, renders these cells particularly vulnerable to endocrine-disrupting chemicals during critical windows of susceptibility. The embryonic and early postnatal periods represent phases of heightened plasticity, during which endocrine-disrupting exposure can induce long-lasting or even permanent alterations in chromaffin cell number, phenotype, and functional capacity.

Thus, endocrine-disrupting chemicals may interfere with chromaffin cell development through multiple mechanisms: (i) direct effects on neural crest cell migration and survival via alteration of guidance cue signaling; (ii) modulation of transcription factor activity through epigenetic mechanisms such as DNA methylation or histone modification; (iii) disruption of glucocorticoid synthesis in the adrenal cortex and signaling; and (iv) induction of oxidative stress, which can impair mitochondrial function and trigger apoptosis in differentiating chromaffin cells [115]. The concept of “developmental reprogramming” suggests that endocrine-disrupting exposure during these critical periods may not cause overt morphological abnormalities but instead induce subtle shifts in the set points of the sympatho-adrenal system, predisposing the organism to altered stress reactivity, metabolic dysfunction, or cardiovascular disease later in life [6].

### 4.3. Affection of Chromaffin Cell Development by DDT

Environmental studies and experimental data did not reveal malformations of the adrenal glands or chromaffin cell ectopia in DDT-exposed animals [96,116]. Developmental exposure appeared to negatively influence the adrenal cortex during postnatal life, which results in adrenocortical hyperplasia [116,117]. Normal anatomy and histology of the adrenals suggest that prenatal exposure to DDT does not affect the formation of adrenal anlage, the migration of neural crest cells, or their transcriptional regulation. Histological evaluation of exposed laboratory animals revealed changes in growth of the adrenal medulla, indicating that the real health risk of low-dose DDT exposure is a shift in the developmental program and altered proliferation patterns [118]. The growth of the medulla during prepubertal and pubertal ages, active in intact rats, is attenuated due to a reduced proliferation rate of chromaffin cells in DDT-exposed animals. Proliferation of chromaffin cells is tightly associated with the expression of hematopoietically expressed homeobox (Hhex), a transcription factor, known as an inhibitor of cell division [119,120,121,122]. DDT-exposed rats demonstrate higher Hhex expression in chromaffin cells during puberty and significant downregulation of Hhex expression after maturation [120]. Suppression of Hhex activates cell division, which in turn compensates for delayed growth but also increases the risk of hyperplasia. The period of ontogeny when exposure begins also matters. Exposure to low doses of DDT since the first day of postnatal life results in more profound changes in proliferation disturbances [96,101,118]. This can probably be attributed to the stage of active differentiation of chromaffin cells, which lasts up to the 10th day of postnatal ontogeny. Since proliferation and differentiation are consequential processes, a higher inhibition of the former may indicate interference of DDT in cell maturation.

Shinomiya and Shinomiya first demonstrated that DDT compromises chromaffin-lineage differentiation at concentrations below overt cytotoxicity [123]. They found that both *o,p’*-DDT and *p,p’*-DDT suppressed differentiation of PC12 pheochromocytoma cells initiated by stimulation with nerve growth factor, implicating p44/42 mitogen-activated protein kinase stimulation in suppressed differentiation. The abovementioned results of our investigations, namely, progredient loss of tyrosine hydroxylase expression in normoblastic chromaffin cells with prolonged exposure to low doses of DDT, suggest probable dedifferentiation of mature cells [95,96]. Dedifferentiation is the biological process where specialized, hormone-producing cells lose their mature traits and revert to a simpler, progenitor-like state that can express stem cell markers and potentially re-enter the cell cycle. Loss of specialization is a key physiological mechanism for self-renewal and maintenance of cell populations during postnatal ontogeny and can also be a target of endocrine disruption.

## 5. Disruption of Chromaffin Cell Renewal

Chromaffin cells have a low capacity for physiological and reparative regeneration; nevertheless, they renew their population throughout life. Current evidence supports three non-exclusive routes by which the chromaffin population may be maintained or restored [104].

### 5.1. Spontaneous Division of Differentiated Chromaffin Cells

The simplest mechanism, supported by the co-localization of proliferation markers with chromaffin antigens, holds that a subset of mature cells retains the capacity for limited self-renewal. The reported data indicate that this route is quantitatively significant chiefly during the neonatal period and becomes minor thereafter [124,125].

### 5.2. Multipotent Progenitors of Glial Type

The most substantial recent advance concerns sustentacular cells—the peripheral, glia-like population long assigned a purely supportive role. Single-cell transcriptomic analysis identified a cluster lacking chromaffin markers but expressing a progenitor signature partly shared with embryonic Schwann cell precursors, including *Plp1*, *Sox10*, *S100b*, *Gfap*, and, previously unreported in this context, *Sox2* [56,125]. Genetic lineage tracing subsequently demonstrated that postnatal SOX2^+^ cells arise as a distinct population from embryonic Schwann cell precursors, persist in the adult human medulla, and generate chromaffin cells of both the adrenaline and noradrenaline lineages in vivo and in vitro [125]. A second paracrine function was also identified: SOX2^+^ cells sustain chromaffin cell proliferation through secretion of WNT6, establishing a direct mechanistic link between the stem compartment and canonical Wnt signaling in the surrounding parenchyma [56,125].

### 5.3. Dedifferentiation of Mature Cells

The third route posits that highly differentiated chromaffin cells can partially reverse their phenotype, downregulating terminal markers and re-entering the cycle. Our findings show that when the adrenal gland completes its growth and cell proliferative activity declines, highly differentiated chromaffin cells alter their transcriptional profile. Along with high tyrosine hydroxylase expression, some cells begin to activate canonical β-catenin/Wnt signaling and the Sonic hedgehog pathway [119]. Canonical Wnt signaling is involved in a plethora of processes, including changes in gene transcription, cell proliferation, migration, and differentiation [126,127]. It is crucial for cell fate determination. The Sonic hedgehog signaling pathway is implicated in neurogenesis during embryonic development and the repair of nervous tissue in postnatal life [128,129,130]. Besides activation of morphogens, adult chromaffin cells also demonstrate upregulation of the pluripotency factor Oct4, which controls maintenance of the pluripotent state [119]. Thus, the adrenal medulla creates a pool of highly differentiated chromaffin cells ready for further dedifferentiation.

Investigations have shown that developmental exposure to DDT disrupts the balance of renewal mechanisms. DDT negatively affects the production of β-catenin, a key mediator of canonical Wnt signaling, and its translocation to the nucleus [131]. Changes in canonical Wnt signaling do not correlate with the altered proliferation pattern of chromaffin cells, whereas the Sonic hedgehog pathway and Oct4 demonstrate close associations with their proliferation rate. Suppression of DDT-induced proliferation of chromaffin cells during puberty is associated with a higher number of Sonic hedgehog-activated cells and Oct4-positive cells, and the concomitant increase in proliferation is related to a smaller increase in Sonic hedgehog- and Oct4-expressing cells [101,131]. Thus, DDT depletes the sources of cell renewal.

## 6. Epigenetic Reprogramming as a Mechanism of Long-Term Effects

One of the most significant advances in endocrine disruptor research over the past decade has been the recognition that exposure to chemicals such as DDT can induce stable epigenetic modifications that persist long after the initial exposure has ceased and may even be transmitted to subsequent generations [132,133]. This phenomenon, termed “developmental reprogramming” or “epigenetic inheritance,” provides a molecular mechanism for the DOHaD concept and explains how transient environmental exposures can produce lifelong health consequences [134].

Epigenetic modifications include three major mechanisms: (i) DNA methylation, primarily at CpG dinucleotides; (ii) histone post-translational modifications (acetylation, methylation, phosphorylation, and ubiquitination); and (iii) non-coding RNA expression, particularly microRNAs (miRNAs) and long non-coding RNAs (lncRNAs) [135]. These modifications regulate gene expression without altering the underlying DNA sequence and can be stably maintained through cell division, making them ideal candidates for mediating long-term environmental effects on gene expression.

Nilsson et al. revealed that the environmental toxicant DDT induced transgenerational epigenetic inheritance of ovarian pathology and granulosa cell epigenome and transcriptome alterations [136]. Sadler-Riggleman et al. demonstrated alterations in critical gene pathways, such as the pyruvate metabolism pathway, in Sertoli cells of a rat F3 generation exposed to DDT. These observations suggest that ancestral exposures to environmental toxicants promote transgenerational epigenetic inheritance of Sertoli cells and epigenetic and transcriptome alterations associated with testicular abnormalities [137]. Thus, DDT causes transgenerational changes in endocrine cells of the female and male reproductive systems. Currently, there is no convincing data on its effect on epigenetic regulation in nervous system cells, which requires further research to elucidate putative shifts in neuronal and glial proliferation and differentiation, the release of neuromediators, and the impact of DDT exposure on previous generations to the recorded increase in incidence of depression and behavioral disorders [138,139,140]. Nevertheless, within the context of the embryonic origins of adult disease, DDT and its analogs represent critical triggers for later-life pathologies. This underscores the need to formally recognize persistent endocrine disruptors as definitive causes of these chronic conditions.

## 7. Integrative Mechanisms of Chromaffin Cell Vulnerability

This review systematically examines the impact of DDT and its persistent metabolites on the development and function of adrenal chromaffin cells, revealing a complex web of molecular, cellular, and epigenetic mechanisms. The key findings are summarized as follows:

*Suppression of tyrosine hydroxylase*. The most consistently reproduced in vivo finding is progressive loss of tyrosine hydroxylase immunoreactivity in the chromaffin cytoplasm, accompanied by the emergence of a tyrosine hydroxylase-negative subpopulation absent from control medullae [101,118]. Because tyrosine hydroxylase catalyzes the rate-limiting step of catecholamine biosynthesis and simultaneously defines terminal chromaffin identity, its depletion is intrinsically ambiguous. This may reflect transcriptional downregulation in otherwise intact cells, failed terminal differentiation of newly generated cells, or dedifferentiation of mature ones. No study has yet distinguished these possibilities by measuring the tyrosine hydroxylase gene transcript alongside protein in exposed medullae, and this remains a central experimental gap. It can be stated that the effect is not secondary to cell death: no significant chromaffin apoptosis or necrosis has been reported at these exposure levels [95,96].

*Depletion of mitochondria and oxidative stress.* DDT cytotoxicity in vitro proceeds through excessive production of reactive oxygen species and depression of superoxide dismutase activity, concomitant with mitochondrial permeabilization [41,42,43]. In vivo, the structural counterpart is matrix swelling, cristae disorganization, and loss of mitochondria with the disproportionate depletion of the subplasmalemmal pool [95,99]. This is mechanistically decisive: subplasmalemmal mitochondria shape the local Ca^2+^ microdomains that gate granule fusion [10]; therefore, their selective loss impairs granule release and contributes to lower catecholamine blood levels.

*Interference with differentiation*. In vitro blockade of differentiation by DDT in pheochromocytoma cells induced by nerve growth factor indicates disruption of paracrine regulation. These findings require in vivo confirmation and further investigation of paracrine stimuli sensitive to endocrine disruption.

*Disruption of developmental and renewal transcription.* Developmental exposure perturbs the age-related dynamics of canonical Wnt/β-catenin signaling, Oct4, and Sonic hedgehog in adrenal tissue and disturbs the transcriptional regulation of postnatal medullary growth and cell renewal [118,131]. DDT exposure evokes developmental delays and insufficient formation of reserves for cell turnover.

**Figure 4:**
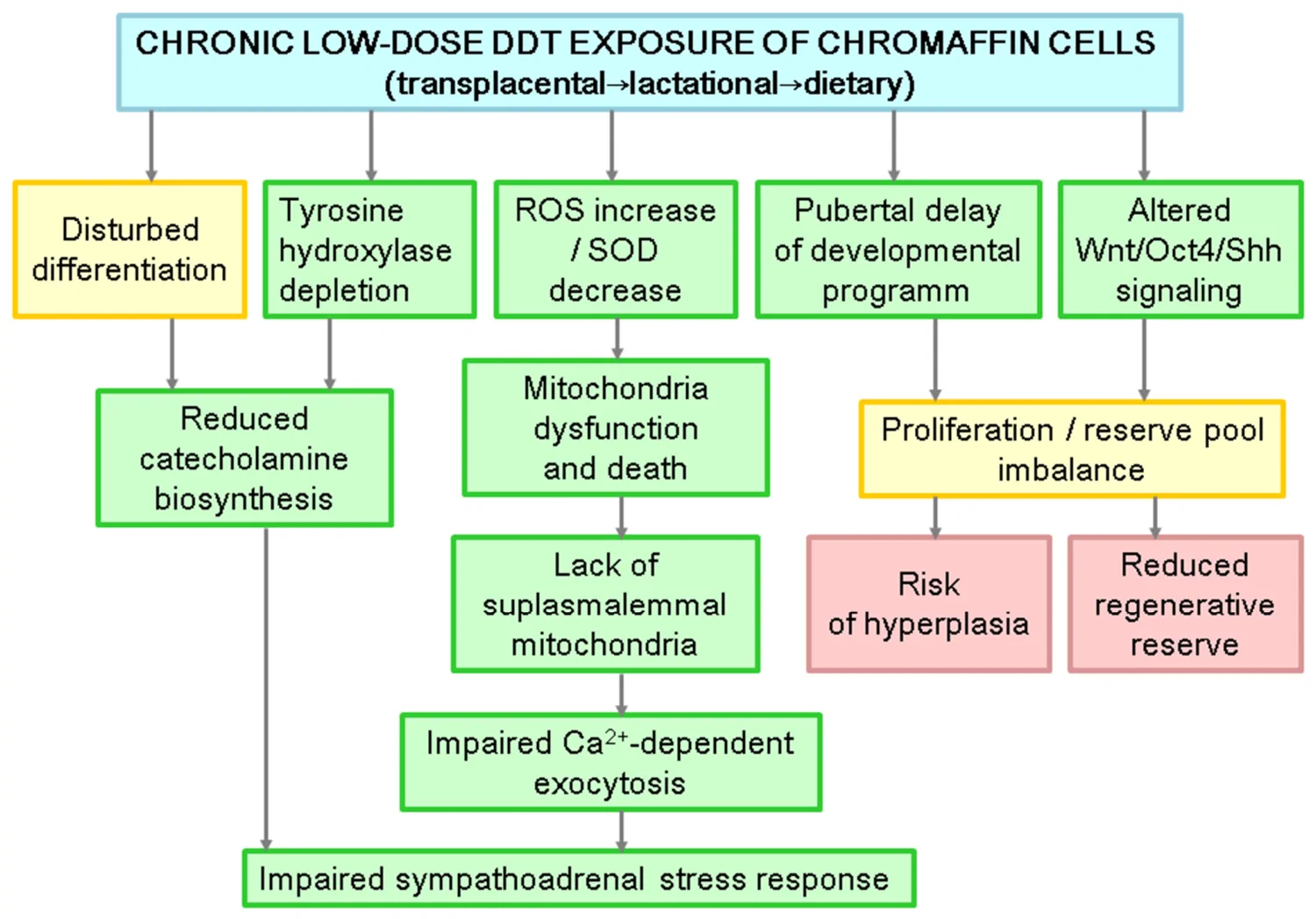
Proposed integrative model linking chronic low-dose DDT exposure to disrupted catecholamine homeostasis. Node color encodes the strength of supporting evidence: **green**, high confidence (reproduced in vivo); **amber**, moderate confidence (supported in vitro); and **red**, hypothesis requiring direct experimental verification. Abbreviations: ROS, reactive oxygen species; SOD, superoxide dismutase; Shh, Sonic hedgehog.

In Figure 4, we present an integrative model of the action of DDT on chromaffin cells, indicating the strength of evidence for certain events and showing the need for research and verification of the in vitro data obtained in animal experiments and emerging hypotheses.

## 8. Knowledge Gaps and Challenges

Despite significant advances in understanding DDT’s disruptive action, several critical knowledge gaps remain.

*Human relevance:* Most mechanistic studies have been conducted using rodent models or cell lines (PC12 and primary CC cultures). The extent to which these findings translate to human adrenal medulla function remains uncertain, particularly given the species differences in adrenal anatomy and DDT metabolism.

*Mixture effects:* Humans are exposed to complex mixtures of endocrine disruptors, not isolated DDT. The potential synergistic, additive, or antagonistic interactions between DDT and other organochlorine pesticides, phthalates, bisphenols, and perfluorinated compounds have largely remained unexplored in the context of adrenal function.

*Sex-specific effects:* Preliminary evidence suggests sexual dimorphism in DDT-induced adrenal effects; however, the underlying mechanisms (hormonal, metabolic, and epigenetic) and their implications for sex-specific disease susceptibility remain poorly understood.

## 9. Future Research Directions

Addressing these knowledge gaps requires a multifaceted approach.

*Longitudinal cohort studies:* Prospective birth cohorts with detailed exposure assessment (maternal and child DDT levels, metabolite profiles) and repeated measurements of adrenal function (salivary cortisol, plasma catecholamines, and cardiovascular reactivity) are needed to establish causal relationships in humans. Integration of epigenetic profiling (DNA methylation and miRNA expression) would help identify mechanistic links.

*Transgenerational studies:* Well-designed multigenerational studies in animal models, with careful control of exposure timing, dose, and route, are essential for elucidating the mechanisms underlying epigenetic inheritance and identifying critical windows of vulnerability. These studies should include both maternal and paternal transmission pathways.

*Systems biology approaches:* The integration of transcriptomic, epigenomic, proteomic, and metabolomic data using network analysis and machine learning could reveal novel pathways and identify key regulatory nodes that could serve as therapeutic targets.

*Intervention studies:* Preclinical studies testing the efficacy of antioxidant therapies, epigenetic modifiers, or lifestyle interventions (diet and exercise) in reversing or mitigating DDT-induced adrenal dysfunction are of direct clinical relevance.

*Wildlife monitoring:* Ecological studies assessing adrenal function and stress reactivity in wildlife populations exposed to DDT and other organochlorines could provide insights into population-level consequences and inform conservation strategies.

## 10. Final Considerations

The adrenal medulla, with its unique developmental trajectory, complex regulation, and critical role in stress adaptation, represents both a vulnerable target and a sensitive indicator of endocrine disruption. The studies reviewed here demonstrate that low-dose DDT exposure, regardless of the onset, whether in the prenatal or postnatal period, can profoundly alter chromaffin cell development, function, and possibly epigenetic programming, with consequences that extend across generations. It is difficult to identify the period of ontogeny most vulnerable to the development of pathological processes in an organ; however, it is clear that exposure to DDT manifests later in life, after puberty. The obtained data indicate the presence of similar changes in sympathetic neurons, which shows the direction of research on the disruptive effect of DDT on the nervous system, including changes in the functioning of synapses. Thus, it creates significant risks of maladaptation and development of somatic diseases. Integrating insights from toxicology, endocrinology, developmental biology, epigenetics, and ecology will be essential to fully understand the impacts of DDT and other endocrine-disrupting chemicals and to develop effective strategies to protect current and future generations from their harmful effects. This opportunity is currently provided by the World Health Organization’s “One Health” concept: an integrated, unifying approach to balance and optimize the health of people, animals, plants, and ecosystems. The legacy of DDT serves as a cautionary tale about the unintended consequences of widespread chemical use and the importance of preventative approaches in environmental policy, agriculture, and food safety. By learning from this legacy and investing in rigorous research, we can better protect the health of humans and ecosystems from the challenges posed by endocrine-disrupting chemicals.

## Data Availability

No new data was created or analyzed in this study. Data sharing is not applicable.

## Abbreviations

The following abbreviations are used in this manuscript:

DBH	Dopamine β-hydroxylase
DDE	Dichlorodiphenyldichloroethylene
DDT	Dichlorodiphenyltrichloroethane
DOHaD	Developmental origins of health and disease
EDC	Endocrine-disrupting chemical
LAT	L-type amino acid transporter
L-DOPA	L-3,4-dihydroxyphenylalanine
PNMT	Phenylethanolamine N-methyltransferase
SNAP	Soluble N-ethylmaleimide-sensitive factor attachment proteins
SNARE	Soluble N-ethylmaleimide-sensitive factor attachment receptor
VMAT	Vesicular monoamine transporter
VGCC	Voltage-gated calcium channel
